# Pilot Federated Analysis Projects

**DOI:** 10.23889/ijpds.v9i5.2515

**Published:** 2024-09-18

**Authors:** Simon Thompson

**Affiliations:** 1 Swansea University

The Cambrian explosion of Trusted Research Environments (TREs) will inevitably lead to increased data siloing and potentially decrease achievable data science. Federated access to data is a mitigation against this risk. The presentation will cover two major infrastructure demonstrator projects to address this need and start the UK journey towards interconnected TRE’s.

TELEPORT was an infrastructure to connect TREs and allow all the data assets to be seen through a single pane of glass while leaving them in situ; the analysis can be performed on each data asset, as well as combining data from multiple TREs to derive knowledge. The analysis is performed within the construct of a pop-up TRE, which is a temporary project-specific TRE. The data governance model is operated in an as-is state, with all egress controls being applied upon extraction of research output. This method has been termed Federated Data.

TELEPORT was an infrastructure to connect TREs and allow all the data assets to be seen through a single pane of glass while leaving them in situ; the analysis can be performed on each data asset, as well as combining data from multiple TREs to derive knowledge. The analysis is performed within the construct of a pop-up TRE, which is a temporary project-specific TRE. The data governance model is operated in an as-is state, with all egress controls being applied upon extraction of research output. This method has been termed Federated Data.

Both solutions were jointly developed by Swansea University / SeRP and share common elements. Future work will see both these products being combined into a hybrid approach as well as spin-out products and services.

